# The Sociodemographic Factors Related to Disability of Applicants of Welfare Benefits in Greece: A Cross-Sectional Survey Based on the World Health Organization Disability Assessment Schedule (WHODAS) 2.0

**DOI:** 10.7759/cureus.55614

**Published:** 2024-03-06

**Authors:** Georgios Theotokatos, Reuben Escorpizo, Theodore J Angelopoulos, Nikolaos K Chrysagis, Aikaterini Venieri, Jerome Bickenbach, Konstantinos Karteroliotis, Eirini Grammatopoulou, Emmanouil Skordilis

**Affiliations:** 1 School of Physical Education and Sport Science, National and Kapodistrian University of Athens, Athens, GRC; 2 Employment and Participation Unit, Swiss Paraplegic Research, Nottwil, CHE; 3 Rehabilitation and Movement Science, College of Nursing and Health Sciences, University of Vermont, Burlington, USA; 4 Laboratory of Advanced Physiotherapy (LAdPhys) Physiotherapy, School of Health and Care Sciences, University of West Attica (UNIWA), Athens, GRC; 5 Sports Excellence, 1st Orthopedics Department, School of Health Sciences, National and Kapodistrian University of Athens, Athens, GRC; 6 Schweizer Paraplegiker Forschung (SPF), Swiss Paraplegic Research, Nottwil, CHE; 7 University of Lucerne, Faculty of Health Sciences and Medicine, Lucerne, CHE; 8 School of Physical Education and Sport Science, Kapodistrian University of Athens, Athens, GRC

**Keywords:** biopsychosocial model, welfare benefits, whodas 2.0, icf, disability assessment, prevalence of disability

## Abstract

Introduction: The aim of the present study was to report on the prevalence of disability and its association with sociodemographic factors among welfare benefit applicants in Greece. The study also compared the disability scores between different health conditions using the WHODAS 2.0 (12-item version), a biopsychosocial-model-based measure.

Methods: The Greek WHODAS 2.0, 12-item version, was administered by interview. A three-member medical committee assessed the medical records of the applicants and assigned a disability percentage based on the biomedical measure of disability percentage determination (Barema scale).

Results: The majority of the participants were female (56.65%). Certain health conditions were presented more frequently among welfare benefit applicants (mental health disorders and neoplasms). The domains with the highest rate of difficulty were the “participation” and “life activities” domains. Significant differences were found between WHODAS 2.0 and Barema scores for all eight different health condition categories. The factorial ANOVA (8x2) showed a significant interaction effect between health condition category and gender with respect to the WHODAS 2.0 score (F = 19.033, p <.001, η2 = 0.13). The WHODAS 2.0 score was negatively correlated to gender, years of studies, and marital status and positively correlated to age, working status, and the Barema score. The results revealed that male participants with a partner who were younger, had more studies, were actively working, and had a lower Barema score would have lower WHODAS scores.

Conclusion: Sociodemographic characteristics of welfare benefit applicants are associated with disability levels based on WHODAS 2.0. Certain health conditions, like mental health or neuromusculoskeletal conditions, are associated with higher disability scores. There are differences between the biopsychosocial and the biomedical approaches to disability assessment. The implementation of WHODAS 2.0 may contribute to a better understanding of the lived experience of patients and is a feasible and efficient tool. Combining biomedical and biopsychosocial approaches may enhance the procedures of disability assessment and help in the development of policies that support people with disabilities.

## Introduction

In 2001, the World Health Organization developed the International Classification of Functioning, Disability, and Health (ICF), which serves as a point of reference and framework for the description of health and health-related status [1]. Based on the ICF, disability includes activity limitations, participation restrictions, and impairments of a person’s body structure and function. In that sense, ICF can serve as a common language between different rehabilitation professionals, facilitate communication, and promote multidisciplinary collaboration [2]. Several validated tools have also been developed based on the ICF for reporting patients’ limitations in functioning: neurological patients (e.g., multiple sclerosis impact profile [3], myasthenia gravis-disability [4], neuromuscular disease impact profile [5]), musculoskeletal patients (e.g., basic mobility scale [6], Whiplash activity and participation list [7]), or other chronic conditions (e.g., functional barometer [8], European child environment questionnaire [9], and the work rehabilitation questionnaire [10]).

The WHO Disability Assessment Schedule (WHODAS 2.0) is directly linked to the ICF [11]. The WHODAS 2.0 examines the functioning level in six domains of daily life: 1) cognition (understanding and communication), 2) mobility (moving and getting around), 3) self-care (attending to personal hygiene, eating, dressing, and staying alone), 4) getting along (interaction with people), 5) life activities (work, school, domestic responsibilities), and 6) participation (community activities and participation in society). The tool is not specific to a health condition but rather considers the generic picture of difficulties in daily life activities. The tool essentially examines how much difficulty people face in given daily activities. There are different versions of the tool of various lengths and modes of administration: long (36 items), short (12 items), self-administered, or proxy versions by interview. The WHODAS 2.0, may be used irrespective of the underlying health conditions and can be administered by healthcare professionals in various settings to enhance clinical practice, research, education, and policy development. In addition, the generic metric that WHODAS 2.0 provides can help inform health and health-related interventions and assess their impact on functioning.

The WHODAS has been translated into at least 47 languages and has been used across different health conditions, study populations, or age groups [12]. Further, it has been used in studies examining the prevalence of disability and the burden of diseases. More specifically, Lee et al. examined the prevalence of disability in the Korean stroke population, utilizing the Korean version of the tool [13]. In another study, the researchers assessed the burden of disability among elderly persons in an urban resettlement colony in Delhi, using the multidimensional concept of disability in WHODAS 2.0. They also explored its association with socio-demographic factors [14,15]. The short form of WHODAS 2.0 has been used in a Spanish elderly population to obtain prevalence estimates of disability levels [16]. Utilizing ICF-based tools, in a Spain-based study, the authors also sought to assess disability in three groups of Spanish primary care patients with stroke, chronic heart failure (CHF), or chronic obstructive pulmonary disease (COPD) [16]. The prevalence of self-reported diabetes and diabetes-related disability amongst older adults has also been examined in South Africa [17]. In the “Wellbeing of Older People Study” in Uganda, researchers used WHODAS 2.0 to describe the prevalence of chronic conditions, their risk factors, and the impact of HIV-related disability on older Africans. In a recent study in Sweden [18], the researchers examined the psychometric properties of the Swedish version of WHODAS 2.0 and also the prevalence of disability in the general population. The WHODAS 2.0 has also been used to evaluate the level of health, functioning, and disability of the population aged 60-70, as well as the analysis of selected social and demographic factors and their influence on disability occurrence in Poland [19].

Our literature review suggested that there were no disability prevalence studies and no respective epidemiological data in Greece examining welfare benefit applicants. There is a lack of epidemiological data on health and disability in welfare benefits in Greece. Assessing the welfare benefit applicants’ functioning by using a tool based on the biopsychosocial model of disability and the ICF adds a holistic view of the disability assessment. WHODAS 2.0 provides the opportunity for health professionals to measure disability using a validated tool that considers the multidimensional aspects of human functioning and, thus, enables a more accurate assessment of needs and tailor-made interventions. Hence, the present study aimed to examine the demographics associated with higher disability rates and the respective health conditions in a wide Greek sample of applicants for welfare benefits.

## Materials and methods

Participants and procedures

The pilot application of the WHODAS 2.0 (12-item, interview-administered) was implemented in the three largest cities in Greece: Athens, Thessaloniki, and Patras. The study started as a pilot project that was initiated in 2018 under the supervision and funding of the Greek Ministry of Labor and Social Solidarity (MoLSS), the World Bank (WB), and the European Commission (EC) Directorate-General (DG) for Structural Reform. The report of the pilot study results was not in the scope of the respective study and can be found in the WB’s report [20]. The MoLSS granted permission for data usage and analysis for a doctoral dissertation. Experts from the WB trained participating doctors who would administer the WHODAS in the guidelines and different scenarios of the administration.

Welfare benefit applicants were adults (≥18 years old) who could speak and comprehend the Greek language (fluent speakers). Non-adult participants were excluded from the analyses. Previous evidence suggests that the WHODAS 2.0 scores reported by the participants tend to reflect higher disability when they seek or receive compensation in comparison to those who have not [21]. Hence, all the participants in the present study were informed that the WHODAS 2.0 results would not affect their entitlement to the welfare benefit under any circumstances. During the disability assessment, a three-member medical committee (consisting of doctors from the public health system) decides on the disability percentage based on the diagnosis and the health records of the applicants. The disability percentage is predetermined and based on the Single Table of Disability Percentage Determination (Barema scale), which dictates the disability percentages for each health condition. For the study, 36 independent medical doctors served as interviewers in three separate centers for assessing individual applications for disability-related welfare benefits. The doctors who implemented the questionnaire were blinded to the health conditions of the participants.

On the same day that WHODAS 2.0 was administered, the applicants were also examined by the three-member medical committee. The three-member medical committees have access to the patients’ medical records and histories, and they attribute disability scores based on the Single Table of Disability Percentage Determination (Barema scale) and the respective health conditions based on the International Classification of Diseases, 10th edition (ICD-10). Historically, the Barema scale has been used to attribute disability percentages to war survivors, veterans, or work-related accidents (e.g., 5% for the amputation of a finger, 50% for the amputation of the lower extremity, etc.) [22]. The Barema disability score also ranges from 0 (no disability) to 100 (totally disabled). 

Health condition, based on the ICD-10, was a variable obtained from the electronic health record of the applicants. There are 22 different health condition categories according to the WHO’s classification [40]. For the analysis of the data, the 22 health condition categories were grouped for classification and better interpretation of the results. This classification was based on the systems that are being affected by the respective disease or injury and lead to similar restrictions, as well as on previous studies that have also used similar categorizations [23-27].

More specifically, the following health conditions were merged: The eye/adnexa (e.g., visual disturbances and blindness) with the ear and mastoid process disorders/conditions (e.g., conductive and sensorineural hearing loss) to diseases of the sensory system (eye/ear) (VII & VIII H00-H59 & H60-H95, respectively) [26], the diseases of the nervous system (e.g., multiple sclerosis, Parkinson disease, etc.) and the musculoskeletal system diseases or injuries (e.g., arthopathies, systemic lupus erythematosus, etc.) (VI & XIII Nervous or Musculoskeletal System [G00-G99 & M00-M99]) [27], the diseases of the cardiocirculatory system (e.g., ischaemic heart diseases, hypertensive heart diseases, diseases of the arteries, etc.) with the diseases of the respiratory system (e.g., asthma, chronic obstructive pulmonary disease, etc.) (IX & X Cardio-respiratory [I00-I99 & J00-J99]) [24], the endocrine/nutritional and metabolic diseases (e.g., diabetes mellitus, obesity, nutritional deficiencies, etc.) with the diseases of the digestive system (e.g., Crohn’s disease, gastric ulcer, vascular disorder of the intestine, etc.) (IV & XI: Affecting Nutrition/Endocrine-Digestive System [E00-E90 & K00-K93]) [23,25]. All the remaining observed conditions were grouped as “other” (e.g., resistance to antimicrobial and antineoplastic drugs, pregnancy or childbirth diseases, poisonings, etc.). In addition, the infectious and parasitic diseases, the conditions that influence health status, and the contact with health services (in the present study, these mainly concerned cases with HIV) were also merged. Based on the ICD-10, patients with HIV are classified with A00-B99 codes, for example, B20: “Human immunodeficiency virus (HIV) disease resulting in infectious and parasitic diseases” or B24: “Unspecified human immunodeficiency virus (HIV) disease." HIV-related conditions are being coded with Z00-Z99, e.g., Ζ21 “Asymptomatic human immunodeficiency virus (HIV) infection status." In this study, Z codes concerned mostly patients with HIV. Furthermore, the mental and behavioral disorders and neoplasms categories were not merged with other health conditions, and they stood alone because of the dominant number of applicants.

Instruments

The WHODAS 2.0 short version (12 items) was used as an outcome measure for disability. The participants were asked the question “How much difficulty did you have in...” the respective daily activities on a 1-5 Likert scale: 1= none, 2= medium, 3= moderate, 4= severe, and 5= extreme or cannot do. For scoring their responses, the answers were recorded, during the data collection, on a 0-4 scale, and the sum was divided by 48 and multiplied by 100 to have a metric where a 0 score is no difficulty and 100 is disabled. This method is referred to as simple scoring because the scores from each of the items are simply added up without recoding or collapsing of response categories; thus, there is no weighting of individual items. When the intellectual capacity of the participant was hindered due to the health condition (e.g., dementia, cognitive impairment, etc.), a proxy version of the questionnaire was used during the interview process, where responses were provided by relatives, caregivers, friends, etc.

The sociodemographic information of the participants was collected through the WHODAS’ 2.0 demographic and background information section (years spent in education, main work status) and other information (age, gender, municipality of application, health condition, etc.) provided by the Ministry of Labor and Social Solidarity, retaining the anonymity of the participants.

Statistical analyses

For the collection and collation of the data, IBM Corp. Released 2021. IBM SPSS Statistics for Windows, Version 28.0. Armonk, NY: IBM Corp. was used. All the descriptive statistics were examined for normality (histogram, skewness, and kurtosis). A factorial one-way ANOVA examined the differences in WHODAS 2.0 scores and the interaction between gender and the different health condition categories. Post-hoc comparisons (Bonferroni correction) were also examined. Multiple regression was used to determine existing associations between sociodemographic factors (an independent variable) and the WHODAS 2.0 score (a dependent variable). A t-test for paired samples was conducted to examine the differences between the two disability scores for each health condition category. The statistical significance of this study was set to p<0.05.

## Results

Participant characteristics

There were 14,113 initial cases. All the non-adult cases were removed (N=1,834). During the process of the medical disability evaluation, applicants who objected to the result were assessed again upon renewal of their appointment. The WHODAS 2.0 in these cases was only administered once, and only the medical result was subject to change. These cases were 398 (duplicate recordings) and were excluded from the analyses. As previously described [28], only complete cases with no missing data or non-applicable items (N=1,718) were included in the analyses. The final sample consisted of 10,163 individuals.

The majority of the cases were female (56.65%). The mean age was 35.13 (±21.20) years (Table 1). About a third (30.55%) had 0-6 years of education (six years are required for graduating from preliminary school in the Greek school system), 43.32% had 7-12 years of studies, and 26.12% had more than 12 years of studies. With respect to the respondents’ living situation at the time of the interview, the majority of the participants lived alone, with family, or with friends in the community (independent living) (80.6%). Only 19.16% stated that they were receiving regular, professional assistance with at least some daily activities, such as shopping, bathing, or meal preparation. More than 40% stated that they were unemployed due to health reasons (N = 4,259), and only 7.98% of the participants were engaged in paid work or self-employed. The majority of the sample was married (42.3%). About two-thirds of the applicants (63.72%) had a single health condition, and the rest had at least two health conditions. The mean WHODAS 2.0 and Barema disability scores can be found in Table 1.﻿

**Table 1:** Sociodemographics of the study’s population.

Variable				N (%)
Gender	Female		5,757 (56.65)	
	Male		4,406 (43.35)	
Education	0-6 years		3,105 (30.55)	
	7-12 years		4,403 (43.32)	
	>12 years		2,655 (26.12)	
Living Condition	Independent in the community		8,191 (80.6)	
	Assisted living		1,939 (19.18)	
	Hospitalized		33 (0.32)	
Marital Status	Never married		3,747 (36.87)	
	Currently Married		4,299 (42.3)	
	Separated		386 (3.8)	
	Divorced		1,094 (10.76)	
	Widowed		543 (5.34)	
	Cohabitating		94 (0.92)	
Work Status	Paid work		578 (5.69)	
	Self-employed		233 (2.29)	
	Non-paid work		30 (0.3)	
	Student		435 (4.28)	
	Keeping house		2,586 (25.45)	
	Retired		497 (4.89)	
	Unemployed (Health-reasons)		4,259 (41.91)	
	Unemployed (Other reasons)		1,423 (14)	
	Other		122 (1.20)	
Number of Morbidities		1	6,476 (63.72)	
		2	2,042 (20.09)	
		3	921 (9.06)	
		4	426 (4.19)	
		5	232 (2.28)	
		6	44 (0.43)	
		7	19 (0.19)	
		8	3 (0.03)	
Version		Non-proxy	8,726 (85.86)	
		Proxy	1,437 (14.14)	
Age (years)		Mean (SD)	35.13 (21.20)	
WHODAS 2.0, 12-item score		Mean (SD)	50.26 (24.26)	
Barema Disability Percentage		Mean (SD)	65.10 (16.20)	

The domains with the highest rate of difficulty were the "participation" and "life activities" domains (2.72±1.01 and 2.45±1.09, respectively). The lowest rating was recorded in the "self-care" domain. The item with the higher rating of difficulty was S5: “How much have you been emotionally affected by your health problems?” (2.98±0.98). The results of the rating for every health condition category can be found in Table 2.

**Table 2:** Average response ratings for every health condition category and the respective domains. Rating of the items was based on the following scale: 1=None difficulty, 2=Mild, 3=Moderate, 4=Severe and 5=Extreme or cannot do. Short version has two items per domain.

Primary Health Condition Category (ICD-10s)	Mobility Mean (SD)	Life Activities Mean (SD)	Cognition Mean (SD)	Participation Mean (SD)	Self-care Mean (SD)	Getting along Mean (SD)
V Mental and behavioral disorders (F00-F99)	1.94 (1.34)	2.75 (0.97)	2.52 (1.09)	2.98 (0.92)	1.23 (1.26)	2.46 (1.16)
II Neoplasms (C00-D48)	2.38 (1.16)	2.41 (0.97)	1.63 (1.14)	2.72 (0.96)	1.13 (1.15)	1.20 (1.15)
I & XXI Infectious diseases or influencing contact with health services (A00-B99 & Z00-Z99)	1.53 (1.35)	1.72 (1.20)	1.18 (1.13)	2.20 (1.08)	0.62 (1.02)	1.05 (1.15)
VI & XIII Nervous or musculoskeletal system (G00-G99 & M00-M99)	2.85 (1.16)	2.80 (0.93)	2.06 (1.19)	2.88 (0.9)	1.79 (1.34)	1.67 (1.34)
IV & XI Affecting nutrition/Endocrine-Digestive (E00-E90 & K00-K93)	1.76 (1.39)	1.91 (1.16)	1.38 (1.21)	2.29 (1.14)	0.73 (1.06)	1.03 (1.19)
IX & X Cardio-respiratory (I00-I99 & J00-J99)	2.77 (1.08)	2.66 (0.99)	1.97 (1.18)	2.89 (0.91)	1.47 (1.31)	1.62 (1.32)
VII & VIII Diseases of the sensory system (H00-H59 & H60-H95)	2.2 (1.4)	2.69 (1.03)	2.28 (1.18)	2.9 (0.93)	1.46 (1.32)	2.03 (1.25)
Other	2.32 (1.29)	2.34 (1.05)	1.49 (1.18)	2.5 (1.02)	1.09 (1.20)	1.17 (1.13)
Total	2.17 (1.34)	2.45 (1.09)	1.90 (1.24)	2.72 (1.01)	1.18 (1.25)	1.65 (1.33)

Clinical characteristics of the participants: multiple comparisons

The majority of the participants’ primary health conditions were mental and behavioral disorders (27.04%), while 21.09% of the sample had cancer as their primary disease. 13.46% had a health condition that was infectious or influenced their contact with health services. 11.99% had a nervous system disorder or a disorder affecting the musculoskeletal system and connective tissues. Overall, 817 participants had as their primary condition a disorder that affects the digestive system or nutrition (8.04%), and 676 participants presented with a disease that affects the circulatory or respiratory system (6.65%). Finally, diseases of the eye/adnexa or the ear/mastoid process were present as a primary disease in 714 cases (7.03%). There were 4.7% of the participants who had some other health condition, so they were grouped as “other.”

In certain diseases, the ratio of males to females was not equal. More women than men exhibited neoplasms (N=1,664 vs. 479), neuro-musculoskeletal disorders (N=884 vs. 335), disorders affecting nutrition (N=500 vs. 317), or diseases of the sensory system (N=434 vs. 280). More men than women exhibited infectious diseases or diseases influencing contact with the health system (mostly HIV) (N=1,061 vs. 307) (Table 3). ﻿

**Table 3:** Frequency count, mean WHODAS 2.0 score, and SD per health condition category.

Variable	WHODAS 2.0 Score	Ν
	Mean (SD)	
*V Mental and behavioral disorders (F00-F99)*	57.84 (22.04)	2748
Males	54.13 (21.65)	1358
Females	61.47 (21.81)	1390
*II Neoplasms (C00-D48)*	47.78 (21.12)	2143
Males	47.83 (21.78)	479
Females	47.77 (20.93)	1664
*I & XXI Infectious diseases or influencing contact with health services (A00-B99 & Z00-Z99)*	34.60 (24.04)	1368
Males	30.17 (22.48)	1061
Females	49.92 (22.96)	307
*VI & XIII Nervous or musculoskeletal system (G00-G99 & M00-M99)*	58.51 (22.07)	1219
Males	53.71 (23.11)	335
Females	60.63 (21.39)	884
*IV & XI Affecting nutrition/Endocrine-Digestive (E00-E90 & K00-K93)*	37.90 (25.04)	817
Males	34.50 (24.19)	317
Females	40.05 (25.36)	500
*IX & X Cardio-respiratory (I00-I99 & J00-J99)*	55.78 (22.71)	676
Males	52.98 (21.78)	343
Females	58.67 (23.31)	333
*VII & VIII Diseases of the sensory system (H00-H59 & H60-H95)*	56.52 (24.64)	714
Males	54.17 (25.01)	280
Females	58.03 (24.30)	434
*Other*	45.47 (22.45)	478
Males	45.96 (23.21)	233
Females	45.00 (21.74)	245

The higher WHODAS 2.0 score was observed in patients with nervous or musculoskeletal disorders (58.51%), followed by mental disorders (57.84%) and diseases of the eye or ear (56.52%). The lowest score was in infectious diseases or diseases influencing contact with health services (34.60%). For the Barema Disability Score, the highest disability percentage was noticed in the diseases of the eye or ear (73.14%), followed by the neoplasms (72.05%), and the cardiorespiratory diseases (65.44%).

There were significant differences between WHODAS 2.0 and Barema scores for all eight different health condition categories. Barema scores were consistently higher in comparison to the WHODAS 2.0 scores (meaning people, as indicated by the Barema score, had more disability than their lived experience). The descriptive statistics (means, standard deviations, and paired sample t-test results) for WHODAS 2.0 and the Barema disability percentage are in Table 4. 

**Table 4:** Frequency count, disability scores, differences based on the WHODAS 2.0, and the Barema Scale for every health condition category.

Primary Health Condition Category (ICD-10s)	Frequency (%)	WHODAS 2.0 Score (Mean [SD])	Barema Disability Score (Mean [SD])	t (p)
V Mental and behavioural disorders (F00-F99)	2,748 (27.04)	57.84 (22.04)	64.05 (11.98)	-13.291 (<0.001)
II Neoplasms (C00-D48)	2,143 (21.09)	47.78 (21.12)	72.05 (15.33)	-45.970 (<0.001)
I & XXI Infectious diseases or influencing contact with health services (A00-B99 & Z00-Z99)	1,368 (13.46)	34.60 (24.04)	57.65 (12.63)	-37.860 (<0.001)
VI & XIII Nervous or musculoskeletal system (G00-G99 & M00-M99)	1,219 (11.99)	58.51 (22.07)	64.88 (16.13)	-9.857 (<0.001)
IV & XI Affecting nutrition/Endocrine-Digestive (E00-E90 & K00-K93)	817 (8.04)	37.90 (25.05)	57.16 (16.42)	-20.239 (<0.001)
IX & X Cardio-respiratory (I00-I99 & J00-J99)	676 (6.65)	55.78 (22.71)	65.44 (16.87)	-9.991 (<0.001)
VII & VIII Diseases of the sensory system (H00-H59 & H60-H95)	714 (7.03)	56.52 (24.64)	73.14 (22.12)	-17.355 (0.001)
Other	478 (4.70)	45.47 (22.45)	63.01 (20.10)	-13.964 (<0.001)
Total	10,163 (100)	50.26 (24.26 )	65.10 (16.20)	-58.245 (<0.001)

One-way ANOVA showed significant differences in the means across the different health condition categories (F = 215.718, p < 0.001, η^2^ = 0.129). Further post-hoc comparisons revealed that the participants who had mental and behavioral disorders did not have significant differences with the patients with nervous or musculoskeletal disorders, or those with cardiorespiratory or diseases of the sensory system. Infectious disorders or disorders that affect contact with the health system did not have significant differences with the disorders affecting nutrition.

**Figure 1:**
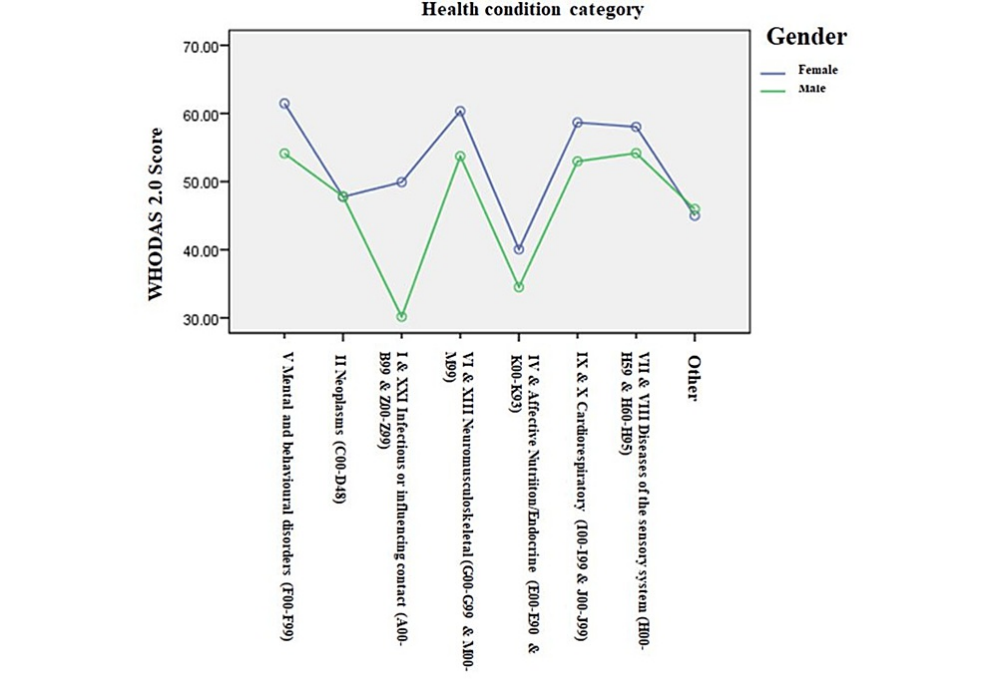
Mean WHODAS 2.0 scores with respect to the gender and health condition categories.

It also examined how the WHODAS score is affected by gender (male or female) and the health condition category (eight different levels). The factorial ANOVA (8x2) showed significant differences for the health condition categories (F = 139.244, p < .001, η^2^ = .088) and the gender (F = 121.434, p < .001, η^2^ = .012). Besides the differences in health condition category and gender, a significant interaction effect was found between health condition category and gender with respect to the WHODAS 2.0 score (F = 19.033, p < .001, η^2^ = .0.13) (Figure 1).

Multiple regression

Multiple regression was used to examine whether the WHODAS 2.0 score could be predicted by the sociodemographic variables. The variables that were included in the model were gender, age, years of studies, working status, marital status, and Barema disability score. The collinearity statistics were in the appropriate range (tolerance ranged from 0.746 to 0.942 and variance inflation factor (VIF) from 1.062 to 1.340), and the model was significant for independent variables that were used as predictors of the WHODAS score (F = 526.292, p < 0.001, adjusted R^2^ = 0.237).

The WHODAS 2.0 score was correlated negatively to gender (B = -2.439, SE = 0.452, β = -0.050, p < 0.001), years of studies (B = -0.666, SE = 0.039, β = -0.155, p < 0.001) and marital status (B = -3.324, SE = 0.467, β = -0.068, p<0.001), and positively correlated to the age (B = 0.437, SE = 0.015, β = 0.297, p < 0.001), working status (B=14.457, SE = 0.791, β = 0.164, p < 0.001) and the Barema score (B= 0.229, SE = 0.013, β = 0.153, p < 0.001). The results revealed that male participants with a partner, and more years of studies, who are younger, with a lower Barema score, and who are actively working would have lower WHODAS 2.0 scores (Table 5). The respective regression equation is as follows:

Y_WHODAS2.0_ = -0,142 - 2,439 * X_gender _+ 0,437 * X_age_ - 0,666 * X_studies_ - 3,324 * X_maritalstatus_ + 14,457 * X_workingstatus_ + 0,229 * X_Barema_

**Table 5:** Regression model summary. Dependent variable: WHODAS 2.0 Score and Adjusted R Square=0.237

Model	Unstandardized Coefficients		Standardized Coefficients	t	Sig.
	B	Std. Error	Beta		
(Constant)	-0.142	2.163		-0.065	0.948
Gender	-2.439	0.452	-0.050	-5.399	0.000
Age	0.437	0.015	0.297	29.646	0.000
Years of studies	-0.666	0.039	-0.155	-16.871	0.000
Marital status	-3.324	0.467	-0.068	-7.120	0.000
Working status	14.457	0.791	0.164	18.273	0.000
Barema score	0.229	0.013	0.153	17.139	0.000

## Discussion

The study aimed to examine the prevalence of disability and the association between disability and sociodemographic characteristics in a wide sample of welfare benefit applicants in Greece. Disability was assessed by the traditional Barema scale and an ICF-based tool: WHODAS 2.0 short form (12 items), which examined the self-reported restrictions that the participants face in their daily lives. The participants presented a variety of health conditions and a combination of different diseases. Certain health conditions presented with higher scores of disabilities according to WHODAS 2.0, and accordingly, the participants had more difficulties and limitations in certain activities. Results showed a significant association between the sample’s characteristics and disability levels, as shown by WHODAS 2.0. In addition, there were significant differences between the WHODAS 2.0 and Barema disability scores.

Epidemiological and clinical characteristics

Similarly, to previous studies, the WHODAS 2.0 disability score (meaning higher disability) was higher among women [16]. Another common finding with previous studies is that female participants, at a higher age and lower education level, exhibited higher WHODAS 2.0 scores [14,18,29]. Previous findings also suggested that age and HIV status were significant variables in predicting disability in older participants in Uganda (above 50 years) [30]. In a Spanish population, WHODAS 2.0 showed higher scores of disabilities in older women with health conditions such as dementia, chronic liver disease, severe mental disease, and stroke [31]. The present findings are also by Ćwirlej-Sozańska and Wilmowska-Pietruszyńska (2018) [19], who found that being with a partner and not being single is linked to less disability.

The working status of people with disabilities can affect positively their health and well-being. When they are employed in a working environment that facilitates accessibility and are offered integrative working training, people with disabilities face less isolation and improve their financial independence [32]. The results of the regression analysis showed that being employed or self-employed is a predictor of lower WHODAS 2.0 scores, meaning less disability. Another finding was that 42% of the sample declared that they were unemployed due to health reasons. This rate of unemployment is indicative of the profile of welfare applicants, while the mean age of unemployed participants was 47.88 (±13.35) years, being of working age.

The disability percentage attributed by the medical committee (based on the ICD-10 and the Single Table of Disability Percentage Determination-Barema scale) was a significant predictor of the WHODAS 2.0 score. The ICF and ICD-10 are two complementary classifications of the World Health Organization, and the utilization of both can help to better understand patients’ restrictions due to a health condition and can enhance the practice and collaboration of health professionals [33]. In a nutshell, the results from the regression suggest the complexity of disability and that it can be affected by several factors, such as age, educational level, working or marital status, etc.

In the present study, the 12-item version was utilized; hence, the results cannot be compared to previous studies that have used the 36-item version. Further conclusions may not be extracted for each domain since only two items are used in the short version for each domain accordingly. Nevertheless, the mean scores of each domain and for each health category have been presented for a qualitative appraisal of the results. The domain with the highest average limitations is the "participation" domain, followed by the "life activities" domain. In previous studies, it was found that the "self-care" domain had lower ratings on the 5-point Likert scale (rated as no problem or mild disability), and the life activities domain showed the highest prevalence of severe or extreme difficulties ratings [16,19].

Despite the fact that the participation domain presented a higher average score, there is heterogeneity between different health conditions and which domain is rated with more difficulties. For example, the “getting along with other people” domain in people with mental health conditions was the second highest-rated domain. In patients with mobility limitations (neuro-musculoskeletal patients), the second highest-rated domain was the "mobility" domain. In patients with neoplasms, conditions affecting the endocrine or digestive systems, and patients with conditions of the sensory system, the second highest-rated domain was the "life activities" domain. It appeared therefore that there may be trends with the most difficulties and certain health conditions. Nevertheless, the association between health conditions and domain limitations is to be done with caution since the short WHODAS 2.0 version was implemented and only two items represented each domain, respectively.

Disabled and non-disabled status

In previous studies, different thresholds have been utilized to classify individuals as "disabled" or "non-disabled" and/or describe the magnitude of health problems and disability as no disability, mild disability, moderate, severe, or extreme disability [16,19]. More specifically, WHODAS 2.0 (36-item version) scores have been categorized as no problem (0-4%); mild problem (5-24%); moderate problem (25-49%); severe problem (50-95%); and extreme/complete problem (96-100%) in terms of ICF categories [16,29,31]. Other authors used the threshold of disability to be 40%, citing the World Report of Disability [34] by the World Bank [14,15]. On the other hand, the World Report on Disability by the World Bank does not mention WHODAS 2.0 as the primary tool. In addition, it is stated in the report that the 50% threshold can also be utilized as a cut-off score for disabled vs. non-disabled status. In the present study, it was decided not to implement the previously used cut-off score for our sample since we used the 12-item version of WHODAS and not the 36-item version. Based on the WHODAS manual, the 12-item version explains 80% of the long version’s variance [11]. It is arbitrary to use a disability threshold based on some other tool’s or version’s results.

Points of reference of the WHODAS 2.0

The WHODAS guidelines provide certain frames of reference for answering the questions. The references are as follows: 1) degree of difficulty; 2) due to health conditions; 3) time frame (the last 30 days); 4) average good and bad days; 5) as the respondent usually does the activity; 6) items not experienced in the past 30 days are not rated. A few comments on these frames of reference that should certainly be taken into consideration are the following:

The third frame of reference suggests that the respondents should rate the items based on how much difficulty they faced over the past 30 days, due to recall abilities that are most accurate for one month. Nevertheless, many of the applicants suffer from flare-ups of their condition; for example, in cases of cancer, patients undergo active treatment like chemotherapy, during which they have tremendous difficulties. At this point, it should be pointed out that the participants in the study have gone through the application procedure long after a major health crisis, surgery, or flare-up takes place. That means that the third frame of reference limits them from reporting the difficulties that they might have experienced a few months ago (with certainly more difficulties).

Another point of reference is that the participants appraise the difficulties they face as they usually do the activity. Hence, if they have assistive devices or personal assistance, they should take this into account. For example, in the case of a tetraplegic person who has a personal assistant that helps with bathing and therefore experiences no difficulty with washing his or her whole body because of the help available, the item would be rated “1” for "None." The interviewers did not evaluate the added value of the personal assistant; hence, they only asked the question once without defining how much difficulty they would have had if they had not had the personal assistance. In addition, in many interviews, it was mentioned that usually, one or more family members quit their jobs to take care of their disabled relative, a variable that is also latent. Informal caregiving creates a financial burden for the family, taking into consideration the unpaid work. The reality that people with disabilities are being helped by other family members or friends is not unique to Greece; it is rather an international phenomenon and is described as a “shadow workforce in healthcare” [35]. As such, it was also presented by Bell et al. (2019), who described the respective circumstances in the United States and suggested the support of policies for people who serve as personal assistants without any recognition by the state [36]. In addition, the participants would have higher disability scores if it were not for a supportive family environment, since only 19.16% of the applicants declared that they hire professional caregivers.

Limitations/Future studies

The categorization of the health condition was based on the electronic health file that the patients submitted during their application to the government. The primary condition, as it appeared through the system, is based on the specialization of the treating physician. For example, if the medical doctor who helped a person with schizophrenia and lung cancer apply for welfare benefits was a psychiatrist, then the psychiatrist would start the procedure, and then he or she would refer the patient to the other treating doctor to add information to the health file. This person’s primary health condition would be registered as a "mental and behavioral disorder" and not "neoplasm." That said, there might be cases where the primary condition is different, but the doctor did not know or could not initiate the health file of the patient (for their own personal or administrative reasons).

Second, WHODAS 2.0 was administered individually, by the physicians, to applicants for welfare benefits in Greece. The measure is subjective, and we may only assume that the patients answered in good faith. We attempted to overcome the above limitation through the validity and reliability results reported in a previous study [37]. The proven use of WHODAS 2.0 in other studies [38,39] on people who applied to Taiwan's disability registration system (more than 150,000 cases) has been proven to be useful for the development of social and health services and policies.

In the respective study, the application of other tests that concern the quality of life, or clinical tests (e.g., handgrip, six-minute walking test, five times sit to stand, etc.) was not feasible. By administering other tests simultaneously, we would be able to compare the results between the different measures and the WHODAS 2.0. A blended measure of self-reported information and performance-based tests to assess functioning might be a good research direction but can be elusive to undertake given its methodological challenges.

The administration of the full version of WHODAS 2.0 was not feasible either. On the day of the assessments, the applicants had to go through the questionnaire and the medical committee’s interview. Hence, it was decided to apply a time-efficient and quick version of the tool so as not to burden the participants.

## Conclusions

The disability of welfare benefits applicants, as depicted by the WHODAS 2.0 12-item version, depends on several factors and is associated with the demographic characteristics of the participants. Higher disability scores were observed in certain health conditions and more difficulties in specific domains of daily life. The implementation of an ICF-based tool such as WHODAS 2.0 may contribute to a better understanding of the restrictions the patients face and is suggested as a feasible and time-efficient tool. A biopsychosocial approach along with a biomedical approach may enhance the procedure of disability assessment of a population and help in the development of policies that support people with disabilities.
